# Supplementary material for: Human genetic ancestry, Mycobacterium tuberculosis diversity, and tuberculosis disease severity in Dar es Salaam, Tanzania
Source: eLife. 2026 Mar 24;14:RP103533. doi: 10.7554/eLife.103533 (PMC13012724; doi:10.7554/eLife.103533)
Supplement: Supplementary file 1. — The latitude and longitude are given in decimal degrees. In the case of two associated regions, a location close to the border of the two regions was selected. [file elife-103533-supp1.docx]

| **Supplementary File 1 - The different ethnic groups with at least 10 members in our cohort and the region and broad geographic location of the original area of the ethinic group.**  The latitude and longitude are given in decimal degrees. In the case of two associated regions, a location close to the border of the two regions was selected. | | | | |
| --- | --- | --- | --- | --- |
| **Ethnic group** | **Region** | **Location** | **Latitude** | **Longitude** |
| Rangi | Dodoma | central | -6.5738 | 36.2631 |
| Digo | Tanga | northeast | -5.06667 | 39.1 |
| Gogo | Dodoma | central | -6.5738 | 36.2631 |
| Hehe | Iringa | southcentral | -7.7681 | 35.6861 |
| Jita | Mara | northern | -1.25 | 34.15 |
| Makonde | unclear | southeast | - | - |
| Nyakyusa | Mbeya | southern | -8.9094 | 33.4608 |
| Nyamwezi | multiple | westcentral | - | - |
| Sukuma | African Great Lakes | northwest | - | - |
| Zaramo | Dar es Salaam and Pwani | eastcentral | -7.3238 | 38.8205 |
| Chaga | Kilimanjaro | northeast | -4.1337 | 37.8088 |
| Haya | Kagera | northwest | -1.91667 | 31.3 |
| Kwere | Pwani | eastcentral | -6.3368 | 38.3939 |
| Ndengereko | Pwani | eastcentral | -7.3238 | 38.8205 |
| Ngindo | multiple | southeast | - | - |
| Pare | Kilimanjaro | northeast | -4.2678 | 37.9315 |
| Pogolo | multiple | central | - | - |
| Shambaa | Tanga | northeast | -4.75 | 38.5 |
| Yao | Ruvuma and Mtwara | southern | -10.922551 | 38.00335 |
| Zigula | northern Pwani, southern Tanga | northeast | -5.77759 | 37.81088 |
| Bondei | Tanga | northeast | -5.42254 | 38.96151 |
| Ha | Kigoma | northwest | -4.8824 | 29.6615 |
| Luguru | Pwani and Morogoro | eastcentral | -7.21488 | 38.35386 |
| Makua | Mtwara | southern | -10.6417 | 39.2376 |
| Matumbi | Lindi | southern | -9.1497 | 38.9877 |
| Mwera | Kilwa district | southern | -9.1497 | 38.9877 |
| Ngoni | multiple | southern | - | - |
| Nyaturu | Singida | northcentral | -6.7453 | 34.1532 |
| Shirazi | Swahili coast | eastern | -5.72573 | 39.29856 |
| Ware | Mara | northern | -1.7754 | 34.1532 |
|  |  |  |  |  |
