# Supplementary material for: Human genetic ancestry, Mycobacterium tuberculosis diversity, and tuberculosis disease severity in Dar es Salaam, Tanzania
Source: eLife. 2026 Mar 24;14:RP103533. doi: 10.7554/eLife.103533 (PMC13012724; doi:10.7554/eLife.103533)
Supplement: Supplementary file 2. — The position is based on the reconstructed reference of the ancestor (Chang et al., 2015) and the derived base indicates the base present in the respective introduction. Intro 1 refers to Introduction 1 within L2.2.1, Intro 5 to Introduction 5 within L4.3.4, Intro 9 to Introduction 9 within L1.1.2, and Intro 10 to Introduction 10 within L3.1.1. [file elife-103533-supp2.docx]

| Supplementary File 2 - Phylogenetic markers selected to identify the Introductions.  The position is based on the reconstructed reference of the ancestor ([79](#_ENREF_79)) and the derived base indicates the base present in the respective Introduction. Intro1 refers to Introduction 1 within L2.2.1, Intro5 to Introduction 5 within L4.3.4, Intro9 to Introduction 9 within L1.1.2, and Intro10 to Introduction 10 within L3.1.1. | | | |
| --- | --- | --- | --- |
| **Lineage** | **Position** | **Derived base** | **Introduction** |
| L4 | 1159734 | A | Introduction 5 |
| L4 | 1502120 | A | Introduction 5 |
| L4 | 2090889 | A | Introduction 5 |
| L4 | 225495 | C | Introduction 5 |
| L4 | 2484751 | G | Introduction 5 |
| L4 | 2624654 | T | Introduction 5 |
| L4 | 3840719 | C | Introduction 5 |
| L2 | 119555 | T | Introduction 1 |
| L2 | 1252462 | G | Introduction 1 |
| L2 | 128469 | A | Introduction 1 |
| L2 | 1547909 | A | Introduction 1 |
| L2 | 1608967 | G | Introduction 1 |
| L2 | 2111284 | A | Introduction 1 |
| L1 | 1348760 | T | Introduction 9 |
| L1 | 1392225 | T | Introduction 9 |
| L1 | 1459473 | T | Introduction 9 |
| L1 | 1479463 | G | Introduction 9 |
| L1 | 1578635 | T | Introduction 9 |
| L1 | 1648566 | A | Introduction 9 |
| L1 | 1750150 | T | Introduction 9 |
| L1 | 1956763 | C | Introduction 9 |
| L1 | 1968814 | T | Introduction 9 |
| L1 | 2189095 | T | Introduction 9 |
| L3 | 1157010 | G | Introduction 10 |
| L3 | 2110074 | T | Introduction 10 |
| L3 | 294514 | C | Introduction 10 |
| L3 | 3221361 | C | Introduction 10 |
| L3 | 3630122 | T | Introduction 10 |
| L3 | 3636991 | A | Introduction 10 |
| L3 | 931240 | T | Introduction 10 |
